# Sustaining Protein Nutrition Through Plant-Based Foods

**DOI:** 10.3389/fnut.2021.772573

**Published:** 2022-01-18

**Authors:** Sapna Langyan, Pranjal Yadava, Fatima Nazish Khan, Zahoor A. Dar, Renu Singh, Ashok Kumar

**Affiliations:** ^1^Indian Council of Agricultural Research-National Bureau of Plant Genetic Resources, Pusa, New Delhi, India; ^2^Division of Plant Physiology, Indian Agricultural Research Institute, Pusa, New Delhi, India; ^3^Department of Biotechnology, Jamia Milia Islamia, New Delhi, India; ^4^Dryland Agricultural Research Station, Sher-e-Kashmir University of Agricultural Sciences and Technology of Kashmir, Srinagar, India

**Keywords:** proteins, plants, nutrition, extraction, sustainability

## Abstract

Proteins are essential components of the human diet. Dietary proteins could be derived from animals and plants. Animal protein, although higher in demand, is generally considered less environmentally sustainable. Therefore, a gradual transition from animal- to plant-based protein food may be desirable to maintain environmental stability, ethical reasons, food affordability, greater food safety, fulfilling higher consumer demand, and combating of protein-energy malnutrition. Due to these reasons, plant-based proteins are steadily gaining popularity, and this upward trend is expected to continue for the next few decades. Plant proteins are a good source of many essential amino acids, vital macronutrients, and are sufficient to achieve complete protein nutrition. The main goal of this review is to provide an overview of plant-based protein that helps sustain a better life for humans and the nutritional quality of plant proteins. Therefore, the present review comprehensively explores the nutritional quality of the plant proteins, their cost-effective extraction and processing technologies, impacts on nutrition, different food wastes as an alternative source of plant protein, and their environmental impact. Furthermore, it focuses on the emerging technologies for improving plant proteins' bioavailability, digestibility, and organoleptic properties, and highlights the aforementioned technological challenges for future research work.

## Introduction

Since the beginning of life, plants have been utilized for human benefits, providing food, therapeutics, wood, fibers, and many others. Moreover, plants were considered the bioproduction system for valuable substances and provide many primary and secondary metabolites having therapeutic effects. Primary metabolites (protein, carbohydrates, fats, and nucleic acid) are the building blocks of life. Besides these, the secondary metabolites are produced by plants to protect them from predators and pathogens, cope with environmental stress, attract pollinators, and work as their defense system (1). Proteins are molecules with great complexity and diversity that play an important role in maintaining the structure and function of the living form (2). Therefore, it is being used for many applications such as medicine, food, and feed.

**Figure 1:**
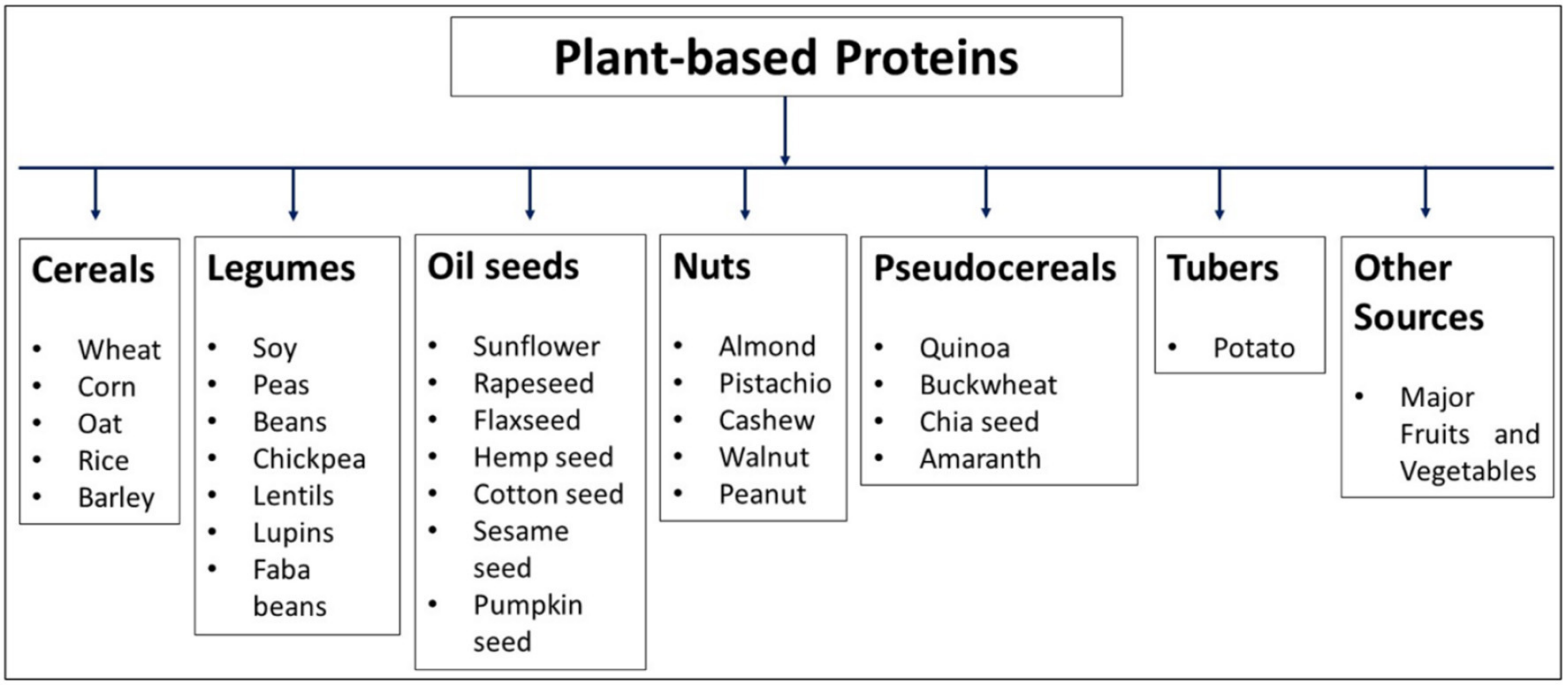
Plant-based proteins derived from different crops.

By 2050, the world's total population is expected to grow or might exceed 9 billion, and, hence, the demand for food, feed, and fiber around the globe is expected to increase by 70% (3). To meet this increasing demand, new sources must be explored. Nowadays, food derived from plants plays a vital role in the human diet as an important source of bioactive components, such as vitamins, phenolic compounds, or bioactive peptides. Hence, these components benefit human health and protect against various disease conditions (4). For meeting protein requirements, generally, animals are considered perfect. However, due to many diseases in animals, their consumption is not safer for human health. Also, it replaces animal-based proteins with plant-based proteins due to various limitations, such as increased cost, limited supply of nutrients, hazard for human health, freshwater depletion, and susceptibility to climate change (5–7). Plant-based proteins are considered vegan food, provide an ample number of amino acids, are directly absorbed by the body, and help in treating various disease ailments. Moreover, the proteins derived from plant-based foods are rich in fiber, polyunsaturated fatty acids, oligosaccharides, and carbohydrates. Hence, they are mainly associated with a reduction in cardiovascular diseases, low-density lipoprotein (LDL) cholesterol, obesity, and type II diabetes mellitus (8). Different sources of plant-based protein that include cereals (wheat, rice, millet, maize, barley, and sorghum), legumes (pea, soybean, bean, faba bean, lupin, chickpea, and cowpea), pseudocereals (buckwheat, quinoa, and amaranth), nuts, almonds, and seeds (flaxseed, chia, pumpkin, sesame, and sunflower) were well-explored (5, 9–11) (Figure 1). However, the demand for the supply of protein is continuously increasing with the rise of the global population (12), hence the need to search for new sources.

It is hard and expensive to extract an adequate amount of animal proteins; therefore, an alternative for improving the nutritional status of humans is mainly received from plant proteins. Hence, attention has been paid to evaluating the nutritional quality of proteins from different plant species. The best way to increase the supply of proteins is to improve the protein expression and efficiency of protein production in natural resources. The advancement of recombinant technologies of protein production, such as engineering of expression hosts, upstream cultivation optimization (e.g., nutritional, bioreactor design, and physical parameters), and development of methods of protein extraction, as well as purification, supported the growth of the market (13). Also, improving the protein functionality in foods through modification, enhancing the plant proteins proportion in human diets, and improving the bioavailability and digestibility of food proteins in the digestion process (14, 15) could be helpful to increase the overall utilization of plant-based protein.

Along with providing amino acids in food, proteins play a significant role in food formulations due to their diverse properties, such as emulsification, gelling, thickening ability, water holding, foaming, and fat absorption capacity (16, 17). Therefore, several thermal techniques (such as cooking, autoclaving, microwave heating, irradiation, germination, fermentation, extrusion, and drying) used during food processing could be optimized to improve the quality of plant proteins (2). Also, they can be isolated from sustainable and cheap sources such as plant-derived wastes from agriculture and by-products of crop and oil industries, which can also regulate food waste reduction (2, 7, 18).

To provide an overview of plant-based protein that helps sustain a better life for humans and the nutritional quality of plant proteins, this review mainly focuses on the current state of using plants to produce proteins for human health. It mainly focuses on various sources and their alternatives with high-quality protein, factors affecting the nutritional value of plant-based protein, bioactivity and functionality, and its modifications. Also, the information on the nutritional quality of proteins derived from plants and potential health issues linked with plant protein will be elaborated. Finally, the issues and challenges of plant-based proteins from availability, consumption, processing, and functionality will be elaborated, and recommendations were made for sustainable production and better utilization of plant-based proteins for meeting human health requirements.

## Plant-Based Sources and Demand of Dietary Protein

### Plant-Based Protein Sources

Among all the existing sources of dietary proteins, plant-based sources dominate the supply of proteins (57%), with the remaining 43% consisting of dairy products (10%), shellfish and fish (6%), meat (18%), and other products from animals (9%) (19).

To provide dietary protein supply and overcome the challenges of feeding the population, several sources of proteins from plants have been searched recently (10, 20–22). Based on sources, proteins from plant origin might lack some essential amino acids. For instance, cereals generally contain less lysine, whereas legumes are deficient in sulfur-containing amino acids like cysteine and methionine (23). However, a good amount of lysine is present in pseudocereals (e.g., quinoa and amaranth). Sometimes, the same plants have different nutrients due to differences in soil diversity, climatic conditions, precipitation levels, geographic latitude and altitude, agricultural practices, and different varieties/cultivars (24, 25). Some traditional plants have been utilized by human beings as protein sources, including beans, pea, and soybean. Also, new sources (such as proteins from insects and algae) (14) and unconventional and alternative protein sources (like agro-industry by-products from the extraction of edible oil and those discarded by fruit processing) have been discovered (7). In addition, different meat, milk, and egg analogs from plant-based protein sources have also been identified (Figure 1) (26).

#### Legumes

A diet rich in legumes provides various health beneficial effects for humans (26). Legumes are considered the best dietary options due to their abundant carbohydrates, protein, energy, vitamins, minerals, and fibers. Various commonly known legume crops for protein and other nutritional sources include soybean, common beans, peas, and chickpea. The protein obtained from soybean has been widely studied (22). Common beans are considered the primary source of vegetable protein in developing countries (27). Highly nutritious legumes such as peas can be utilized for different food product formulations to improve the human intake of protein. Food products from chickpea are the major dietary protein source of high-quality protein (22). The protein isolates and defatted flour from lupin fulfill the requirements of essential amino acids (28). Moreover, pigeon pea and its derived isolates of protein are the potential sources rich in sulfur-containing amino acids suitable for the consumption of human beings (29).

#### Cereals

Cereal consumption, such as wheat, rice, barley, and corn, are the most common staple food throughout the world (30). Globally, in developed and developing countries, rice is one of the most widely consumed cereal crops. Amagliani et al. (30) analyzed the amino acid composition of proteins present in the rice and found that lysine content is highest in albumin, while sulfur-containing amino acids are majorly present in the globulin. Some studies have also been conducted to improve rice protein's extracted yield by using different isolation techniques (31). In one of the studies, it has also been found that lysine is present in significantly less rice protein isolates (32). Mainly consumed in developing countries, millet, and its concentrates of protein are a mostly nutritious source of proteins. It usually contains a high amount of essential amino acids, including lysine. Nutritional profiles of cereal-based proteins have also been extensively used in industrial applications and bakery products. In a study, faba bean flour, and wheat flour bread products showed an increased amount of essential amino acids after fermentation. The mixture of legumes and cereal helps improve the overall nutritional quality (33).

#### Pseudocereals

Pseudocereals like amaranth, buckwheat, and quinoa are mainly the dicotyledonous plants that are considered false cereals (34). Recently, more interest has been paid to utilize pseudocereals protein, like amaranth and quinoa, to fulfill the high demand for proteins. These sources mainly contain high-quality protein, unsaturated fatty acids, fibers, vitamins, and minerals. They also have a high quality of essential amino acids and increased bioavailability of proteins. Along with these qualities, they are also gluten free, being an alternative in the diet of patients with celiac disease (35). One of the studies also showed that amaranth and quinoa contain a high quantity of lysine, useful as dietary supplements (35).

#### Seeds

The consumption of plant-derived food components increases continuously, and seeds are an important source that provides good quality of nutrition (30). Flaxseed, one of the richest sources of high-quality protein, also contains phenolic compounds, fibers, and essential amino acids; however, some studies argued that lysine is limiting in flaxseed (36). In their study, Lugo et al. (37) observed that the composition of essential amino acids in chia lacks lysine, whereas the watermelon seeds were found to contain a good amount of leucine and arginine (38). One of the studies has also been identified that the flour of paprika seed mainly contains aromatic amino acids like threonine, lysine, and tryptophan but poor in sulfur-containing amino acids and isoleucine (39).

#### Almond and Nuts

Almonds and nuts are generally known for their high-quality lipid and fatty acids content and also contain high-quality protein content. The species known as pequi and baru from Brazilian Savanna are non-traditional almonds that are good protein sources and have a complete profile of amino acids (40). Baru almond contains all essential amino acids, whereas pequi almonds are rich in sulfur amino acids and lack lysine, similar to cashew nut (*Anacardiumothonianum*). Peanuts are limited in valine and lysine and are considered as the inferior source of protein.

#### Meat Analogs From Plant Proteins

Currently, commercial plant-based meat analogs revolutionize the modern food industry. In the US, the market price of plant-based products was ~$940 million in 2019, which will increase by 38% in recent years (41). Currently, the food industry helps produce high-quality plant-based meat analogs, such as sausages, burgers, ground meat, and nuggets. However, it is more challenging to make the products that match the properties of whole muscle tissues like connective tissue, muscle fibers, and adipose tissue that form hierarchical structures (42). The arrangement of tissue structure plays a significant role in determining meat products' sensory and physicochemical properties. Plant-based whole muscle products of high quality first require the most suitable ingredients and processing techniques to stimulate muscle fiber, adipose, and connective tissue.

Many reviews have been published on meat analogs from plant proteins (41–44). Ideally, meat analogs should provide adequate structural similarity besides nutrient composition. Meat analogs are mainly produced from plants' macronutrients, including polysaccharides, proteins, and fats, and some micronutrients and other ingredients, such as minerals, vitamins, flavoring, and color agent preservatives, and binders. The components and processing techniques utilized to produce these analogs should be optimized for each meat product. The appearance of the meat analogs' surface should be of opaque texture like real meat. Food industries have used several techniques to maintain the color of plant-based meat alternatives. For instance, Meat™ uses beet juice extract that contains a natural pigment called betalain to recreate the suitable color of meat. Also, Impossible Foods™ uses leg hemoglobin (plant-based heme protein) extracted from soybeans roots to color its products. Various technological and scientific methods, like processing and physicochemical approaches, are being searched to create potential structures of plant-based meat that aim to accurately mimic the texture of real meat. It should also be noted that meat analogs usually simulate the fluid-holding capacity like real meat during cooking. Knowledge about the essential constituents of flavor present in products of real meat is helpful to identify plant-based ingredients that give the meaty flavors in plant-based meat analogs. However, developing plant-based meat analogs is challenging and providing a similar nutritional profile to real meat.

#### Milk Analogs From Plant Proteins

One of the most consumed food products from plant origin is plant-based milk analogs. Various attributes, such as processing methods, sensory quality, raw materials, physicochemical properties, and nutritional profiles of plant-based milk analogs, have been presented and described in many articles (45, 46). Milk analogs are colloidal dispersions consisting of several particles, such as fat droplets, oil bodies, plant tissue fragments, protein aggregates, and insoluble calcium carbonate particles dispersed in an aqueous solution containing soluble proteins, sugars, salts, and polysaccharides (46). For the formation of high-quality milk analogs, there should be correct information on light scattering theory, techniques of particle reduction, as well as mechanisms of particle instability. Two approaches have been used for producing milk analogs, such as disruption of plant tissue (including soaking, mechanical disruption, enzymatic hydrolysis, separation, formulation, homogenization, and thermal treatment for breaking of plants materials into small particles) and homogenization (including blending of plant-based components that are isolated, such as emulsifiers, oils, and thickeners) (46). The components and processing techniques are generally optimized for creating milk analogs that mimic cow's milk's functional and desirable properties (46). For developing a better quality product, the plant-based milk analogs have been extensively analyzed for features, such as appearance, flavor, color, bio-availability, and nutrition profile.

#### Egg Analogs From Plant Proteins

The hen's egg consists of 75% water, 12% proteins, and 12% lipids. Also, it includes a variety of constituents that help in different food applications like foaming, emulsification, and gelation (47). Eggs are mainly used in various ways, such as boiled, fried, poached, or scrambled, and part of many other foods, including dressings, mayonnaise, desserts, and baked goods. Generally, plant-based egg analogs should have desirable functional and physicochemical properties. For example, eggs analogs should have the functional ability to transform a solution into a gel when heat is supplied, just like that of real eggs. Plant proteins used in egg analogs have solution temperature in the range of 63–93°C, which shows that higher temperature is needed to mimic the structure and texture of real eggs. Various methods, such as dynamic shear rheometry and differential scanning calorimetry, have been utilized, which provide information on gelation temperatures and denaturation of proteins. The gel nature of plant-based egg analogs depends on the type of protein (e.g., chickpea, pea, sunflower, bean, and soybean), the concentration of protein, and environmental conditions (e.g., pH, ionic strength, and thermal history). Plant-based egg analogs should have the best emulsifying solubility, segregation, separation, and stabilization properties. Like real eggs, they also have a better appearance, flavor, color, bioavailability, and a nutrition profile to produce a better quality of plant-based milk analogs.

#### Food Waste/By-Products as a Protein Source

Increasing population and industrialization also negatively affected the environmental conditions. Eco-innovation is the term that addresses the essential changes for sustainable development. It is an approach where by-products and waste from plants become an important resource. Food waste/by-products have also been utilized for the extraction of proteins. These mainly include oil meals/press cakes, by-products of cereals, and legume processing.

##### Oil Meals/Press Cakes

During oil processing, the by-products, such as oil meals/press cakes, have been released from oil-bearing fruits and seeds (48). Oil meals contain 15–50% of protein content and are, hence, considered valuable sources for the extraction of proteins (48). Soybean, cottonseed, peanut, sunflower seed, sesame seed, pumpkin seed, hazelnut, grape seed, walnut, hemp seed, and rapeseed are the major oilseed crops containing a high proportion of protein meal. Also, oil-bearing crops, such as coconut, palm, and olive, have oil in their fruit pulp, and their residues are useful to isolate proteins. The protein content varies depending on the processing of hulled and dehulled meals of oilseed. Usually, the dehulled meals have higher protein content and lower fiber content, while dehulled meals require an additional fractionation step before they have been used for protein extraction.

##### By-Products of Cereal and Legume Processing

By-products after cereal and legume processing are important raw materials for the extraction and isolation of proteins. The high content of protein in legumes makes them most important, followed by cereals. Rice bran is the most important protein source among cereals. Along with the rice, several other crops by-products have been used as promising protein sources, such as wheat bran, broken rice, brewer's spent grains, and defatted wheat germ. Commercial milling of pulses also produces ~25% of by-products consisting of powder, husk, broken, shriveled, and unprocessed seeds. With high nutritional value and a well-balanced profile of amino acids and also various bioactivities, the cereal crops and their by-products are of major attention. Hence, these are considered as appropriate materials for protein extraction due to their quantities, availability, and composition of amino acids (30).

### Demand of Dietary Protein

Proteins are molecules with great complexity and diversity that have played an important role in maintaining the structure and function of living cells (29, 49). It is being applied in a number of applications, such as medicine, nutraceuticals, industries, food, feed, etc., and the demand for protein is continuously increasing with the rise of the global population (12). Globally, protein requirements are fulfilled by both plants (80%) (such as cereal grains, beans, soy, pulses, nuts, vegetables, and fruits) and animals (~20%) (such as meats, milk, eggs, fish, yogurt, and cheese) (50). Along with the increasing nutritious food demand, the protein demand is continuously increasing globally by changing socioeconomic status. Increased urbanization, as well as economic development, has led to various transitions in dietary patterns in the population of low- and middle-income countries, especially the demand for foods derived from animals, which is noticed in developing countries (51). Protein from animal origin causes emissions of greenhouse gases from livestock as well as loss of terrestrial biodiversity by human interventions (52). Therefore, plant-based protein requirements are continuously increasing.

Plant-based proteins play a major role in the human diet as they are rich in a large number of other nutrients, vitamins, and minerals (53). Foods obtained from plants enhance the content of protein that contains various essential amino acids and may also improve the nutritional status of human diets. From the last few decades, interest has been drawn for the search of protein sources with high nutritional quality and functionality and industrial applications (like emulsification, solubility, gelation, foaming, viscosity, oilholding, and water-holding capacities). Furthermore, the development and utilization of novel techniques of food processing enhance the nutritional quality of traditional sources of plant protein. According to the overall status of health, human nutrition is considered an important issue that provides the methods for prevention or development of a number of diseases resulting from excessive, unbalanced, or insufficient nutrient intake (15). Generally, the daily intake of protein is provided by animal-based foods. However, changes in the consumers' requirement led to adoption of alternative sources of proteins for human consumption. And, also, the protein produced from animal sources is costly and environmentally non-sustainable and requires more water (about 100 times) during production than plant protein. Emerging factors in animal proteins, like the growth of world population, climate change, and occurrence of animal diseases, more research is now dedicated to finding various new sources and technologies to produce proteins from plants with high content and resilient to changing climate and thus provide balanced nutrition in humans' diet (51).

The proteins and their amino acid composition play a major role in human health. For instance, sulfur-containing amino acids, such as methionine and cysteine, play a vital role in maintaining the immune system functioning (54) and also the peroxidative protection mechanism in muscles, nervous, and cardiovascular systems (55). Lysine is important for bones calcification, liver activities, nitrogen balance inside the body, and muscle and blood synthesis. While valine helps in the coordination of motor cells, and aspartate and glutamate are essential for hormonal regulation and immunological stimulation, respectively (35). Leucine and isoleucine are assisting as building blocks of other proteins (36). Generally, it has been recommended that, for adults, the protein intake should be in-between 0.8 and 1 g/Kg body-weight/day (56, 57). Pregnant, lactating women, and infants need higher protein ingestion than adults as 1.1, 1.3, and 1.2–1.52 g/kg/day, respectively (57). The intake requirement of proteins and amino acids is determined by various factors linked with genotypic as well as phenotypic characters (age, gender, body weight, lifestyle habits, physical exercises, health conditions, and metabolic capacities) (5).

## Factors Affecting the Nutritional Value of Plant Proteins

**Figure 2:**
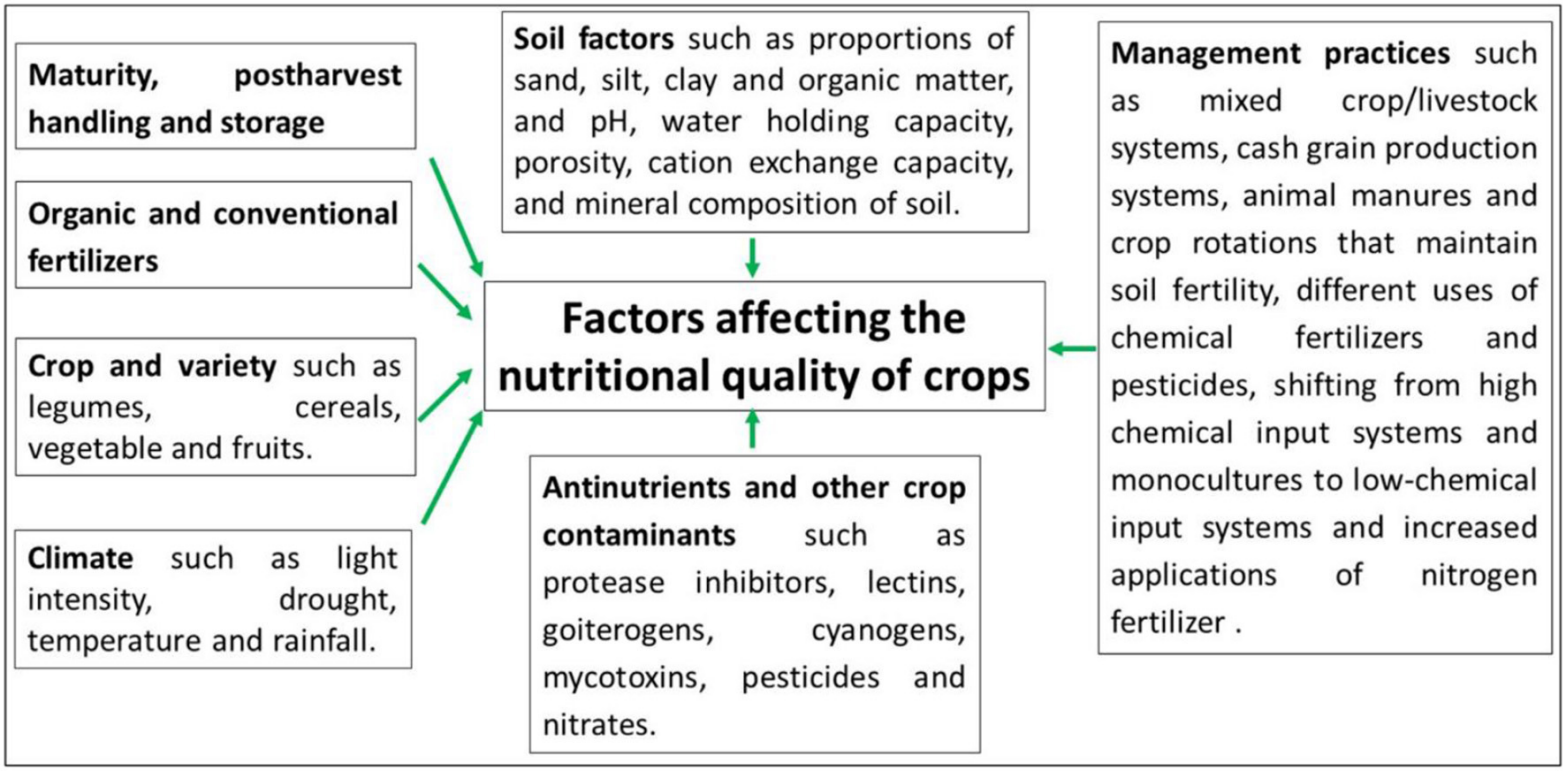
Various factors that affect the nutritional quality of crops.

The protein's nutritional quality can be identified in different ways, but, in a simple way, it is the balance and relative amounts of essential amino acids, as well as digestibility, bioavailability, and bioactivity, which mainly identify its nutritional value. Compared with animal-based protein, the proteins derived from plants are easier to produce; however, when utilized as dietary sources for human consumption, most of the plant proteins are deficient in essential amino acids and are, therefore, nutritionally incomplete. For example, some cereal proteins are low in tryptophan, lysine, and threonine content, while vegetable proteins and legumes have a lower amount of sulfur-containing amino acids, such as methionine and cysteine (58). Due to this deficiency, these essential amino acids become the limiting factor in legumes and cereals. Practically, neither legumes nor cereals can compensate for the deficiency of amino acids for other crops, and, hence, diet feeding regularly provides supplementary amino acids. There are also other factors that affect the nutritional quality of crops, including soil condition, crop maturity, postharvest handling, storage, use of fertilizers and pesticides, crop variety, and climatic conditions (Figure 2).

It is important in terms of nutritional as well as economic value to increase the essential amino acids content in plant-based proteins (59). In the past decades, plant breeders and geneticists have done much research for the improvement of the quality and characteristics of plant proteins. For instance, natural mutations, like the high content of lysine in barley and corn, have been recognized and made as elite genotypes (60). But, unfortunately, undesirable characters, like lower yields and susceptibility to pests and diseases, were also linked with these types of natural mutations. Nowadays, the techniques of modern biotechnology as alternative methods help to solve these problems. The method known as the protein digestibility-corrected amino acid score (PDCAAS) is an effective tool for the quality evaluation of protein (49, 61). One of the new methods recommended by FAO in 2013, digestible indispensable amino acid score (DIAAS), has also been used to evaluate protein quality, and, in terms of scientific knowledge, it is considered more accurate than PDCAAS (62).

## Bioactive and Functional Properties of Plant-Based Proteins

### Bioactive Properties of Plant-Based Proteins

**Figure 3:**
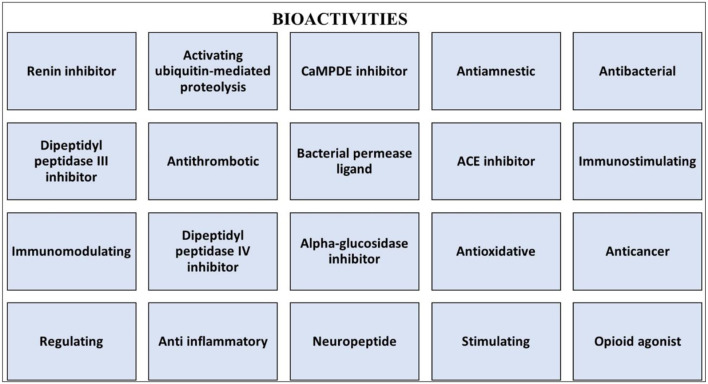
The major bioactivities of plant-based proteins.

Several reports have shown the health effects of plant-based proteins as antitumor, antioxidant, hypoglycemic, ACE inhibitory, antimicrobial, and hypolipidemic effects (Figure 3) (63, 64). It has been observed that in countries where a high number of pulses are consumed, risk diseases, such as type-2 diabetes, cardiovascular diseases, colorectal cancer, and different types of chronic diseases, have been reduced (65, 66). The bioactivity of small peptides that are mainly released from enzymatic hydrolysis by various proteases, such as pepsin, trypsin, chymotrypsin, alcalase, papain, pancreatin, thermolysin, and flavorzyme, are present in different pulse proteins (67). These peptides exert various bioactivities, such as antioxidant, antifungal, antitumoral, and ACE inhibition activity (67, 68), and are also used for different purposes, like food supplements, functional food ingredients, and nutraceuticals (63) (Table 1).

**Table 1:** The commonly used physical modification methods of protein and their applications.

**Modification methods**			**Description/Applications**	**References**
Physical modification	Heat treatment	Conventional thermal treatment	Physically modifying plant-based protein structural and functional properties	(69)
			Used in pharmaceutical and food industries	
			Increase thermal stability, gelling properties	
			Reduce or eliminate adverse effects of anti-nutritional compounds	
			Improve digestibility, nutritional, and emulsifying properties of plant-based proteins	
		Ohmic heating	Used for milk pasteurization	(70)
			Result in unfolding, denaturation, and the formation of uniform-sized protein aggregates	
			Having techno-functional properties	
			Decreasing the heating time and improving the emulsifying ability of the protein	
		Microwave heating	Induce protein unfolding by splitting disulphide and hydrogen bonds	(71)
			Improve digestibility, gelling and emulsifying properties	
			Modulate the protein without destroying its primary structure	
			Increase the efficiency of enzymatic modification	
			Used for immunomodulation of different plant-based proteins	
		Radio frequency treatment	Increasing the surface hydrophobicity	(72)
			Improve functionalities such as the oil holding capacity and emulsifying properties	
		Infrared irradiation	Increase digestibility	(18)
			Decrease the amount of anti-nutritional factors	
	Gamma irradiation		Extending the shelf-life of food products	(62)
			Increase thermal stability, digestibility, and surface hydrophobicity	
			Improve antioxidant ability, oil binding capacity, emulsifying, and foaming properties	
			Decrease water binding capacity and immunoreactivity	
	Electron beam irradiation		Sterilize food materials as well as assist in extraction processes	(73)
			Improve solubility, nutritional value, bioactivity, functional properties, and emulsifying activity of peptides	
			Decrease surface hydrophobicity and molecular weight, which positively affected emulsifying and foaming properties	
			Increased efficiency, antioxidant ability, and thermal stability of this plant-based protein	
	Ultraviolet radiation		Induce chemical modification in plant-based proteins which can improve their techno-functional properties	(74)
			Improve the mechanical properties of the formed films	
			Increase sulfhydryl content, surface hydrophobicity, antioxidant activity, solubility, emulsifying and foaming properties	
			Decrease immunoreactivity and allergenicity of plant-based proteins	
	Pulsed-electric field		Increase surface hydrophobicity, antioxidant activity, protein solubility, emulsifying, foaming and functional properties	(75)
	High pressure treatment	High hydrostatic pressure	Inactivate microorganisms, changes texture and emulsification	(76)
			Increase protein surface hydrophobicity and nutritional value of plant-origin proteins	
			Reduce allergenicity	
			Improve techno-functional properties, gelation and aggregation ability, antioxidant activity, emulsifying properties, protein solubility, colloidal and heat stability, water, and oil holding capacity	
		Dynamic high-pressure fluidization	Inactivate microbial cells	(77)
			Improve emulsifying ability, versatility, digestibility, and functionality of plant-based proteins	
			Decrease nanoparticle size and allergenicity of different animal proteins	
			Increase solubility, emulsifying and foaming properties	
	Sonication		Favor water and oil binding capacity, emulsifying and gelling properties	(78)
			Improve solubility, foaming capacity, emulsification properties, antioxidant ability, and digestibility of plant-derived proteins	
			Enhance hydrophobicity	
			Decrease foaming stability, amount of anti-nutrients, and allergenicity	
	Extrusion		Inactivate microorganisms, enzymes, and naturally occurring toxic substances as well as gelatinization of starch or shaping food materials	(79)
			Improve techno-functionality and digestibility of plant-based proteins, and can also generate a texture mimicking that of meat	
			Destroy anti-nutrients compounds	
	Ball mill treatment		Ensure higher solubility	(80)
			Improve gelling properties	
	Cold atmospheric plasma processing		Inactivate microorganism, spores and viruses present on the surfaces of food	(81)
			Improve techno-functional properties of plant-based proteins, solubility, emulsifying and foaming ability, water holding capacity, antioxidant, and gelling properties and surface activity	
			Increase surface hydrophobicity	
	Ultrafiltration		Improve of surface hydrophobicity, emulsifying, foaming, and oil holding capacity	(82)
			Remove anti-nutritional compounds	

#### Plant-Based Proteins Against Cardiovascular Disease and Metabolic Risk Factors

A large number of studies showed the potential impact of dietary proteins derived from plants against cardio-metabolic risk factors. The first study for the synthesis and intake of plant proteins as an alternative to animal protein was reported and published in 2017 (83). In this study, the authors reviewed and demonstrated biomarkers for cardiovascular disease from plant proteins consumption (83). They also studied and reported a decrease in the concentration of blood lipids (such as lower apolipoprotein B, low-density lipoprotein cholesterol, and non-high-density lipoprotein cholesterol). The authors also conducted randomized trials, which proved that plant protein is effective in reducing the risk factors associated with cardiovascular diseases in adults. In another study, the impact of proteins derived from plants (mostly soy products) on hypercholesterolemic patients was found superior in lowering the lipid profiles compared with the animal proteins (84). In populations, the adolescent stage, most of the benefits of plant-based proteins and metabolic health concerns have been discussed. Several studies to examine the benefits of plant-based proteins intake have been done for metabolic syndrome, weight management, and obesity, as these are the serious and growing health issues globally among adolescents. However, the regulation of protein intake is critical to many physiological development and functions. Therefore, enhancing the proteins derived from plants in adolescent diets as a substitute for animal-based proteins helps in controlling obesity and other cardio-metabolic factors (85). The authors in different studies concluded that there should be the addition of more proteins of plant origin in the human diet for reducing the risks associated with cardiovascular disease as well as metabolic risk factors (86). Also, it was found that consumption of plant-based proteins lowers blood pressure in patients with hypertension (including elderly patients) as compared to animal protein (87, 88).

Most of the studies were also associated with the intake of plant protein sources and mortality. In a recent cohort from the NIH-AARP Diet and Health Study, the authors also observed the effect of choice of dietary protein on mortality (89). In this study, more than six lakh individuals from the U.S. in the age group of 50–71 years were followed from 1996 till December 2011. It was noticed that plant protein intake has led to inverse the mortality rate as well as from stroke in both males and females and cardiovascular disease. They observed the replacing of animal protein with only 3% of plant protein reduced 10% risk of overall mortality in both males and females (89). Therefore, it is beneficial to substitute plant proteins into the diet instead of animal proteins in terms of mortality and longevity. In a recently published review of the 32 cohort studies, it has been interpreted that the plant-based protein diet lowers the risk of all-cause and cardiovascular-associated mortality. Replacement of foods containing animal proteins with plant protein improves longevity (90).

#### Plant-Based Proteins and Diabetes

Although plant-based diets are mainly linked with reducing the risk of diabetes (91), it is still not clear that substituting the plant-based proteins for animal proteins helps in reducing the risk of diabetes in the population. After studying and analyzing using the dataset from the Nurses' Health Study II, Malik et al. (92) observed that 5% substitution of vegetable protein for animal protein was linked to the 23% reduction in type 2 diabetes risk. In a meta-analysis conducted in 2015, the sources of animal protein were replaced with plant-based protein for ~35% of the intake of dietary protein for 8-week randomized controlled trials. From this study, the authors found that there are significant improvements in the levels of fasting glucose, fasting insulin, and HbA1c in patients with diabetes (individuals with both type 1 and type 2 diabetes) (93). In a cohort study, individuals were provided a protein-based diet and found that higher protein intake is associated with a lower risk of diabetic and pre-diabetic incidences, and plant-based proteins are the main determinant (94). The plant-based protein diet also contains a variety of bioactive components, which provide beneficial health effects as compared to processed meat products. In another randomized crossover trial, substituting red meat with legumes (lentils, chickpeas, peas, and beans) significantly decreased fasting blood glucose, insulin, and the triglyceride level in patients with diabetes type-2, suggesting the potential role of plant-based proteins over animals (95).

#### Plant-Based Proteins Against Cancer

Generally, a large number of factors, such as environmental, genetic, dietary, and other habitual features, are associated with the development of cancer. One research group has studied and examined the risk factor of colorectal cancer in individuals with the help of analyzing gene-environment interaction, including other factors, such as genetic, lifestyle, and cancer risk factors (96). The authors reported the linkage between colorectal cancer and the genetic diversity of fatty acid metabolism, which are mainly associated with a higher intake of meat, and concluded that those who consume a high diet of meat have a high risk of colorectal cancer (96). Therefore, plant-based protein substitution for animal protein is a better way to reduce the risk of colorectal cancer in humans with certain genetic polymorphisms.

#### Plant-Based Proteins and Their Renoprotective Effect

The diet, which is lower in vegetables, fruits, healthy oils, and dairy food, but higher in total protein foods, total grains, saturated fats, sodium, and added sugar, has been under trials to know the differences that help to cure chronic disease, especially chronic kidney disease (CKD) (97). Recent studies have suggested that, along with the amount of protein, protein's origin (for example, plant vs. animal) might be a crucial factor that affects the function of the kidney (98). For individuals with chronic kidney disease, on the consumption of plant-based protein, a significant 23% lower mortality rate was reported (99). In a randomized control trial in diabetic adults with macro-albuminuria, the animal protein diet was substituted with soy protein diet (by 50%) and found that it significantly improved proteinuria, cholesterol, and the glucose level (100).

In a crossover study, a diet rich in soy protein reduces glomerular hyperfiltration in individuals having type 1 diabetes with early-stage nephropathy (101). With the increase in glomerular hyperfiltration and the glomerular filtration rate, the incidence of kidney injury has been decreased (102). The plant-based proteins mainly extracted from rice endosperm and soybean have also shown renal protective function in diabetic rat models (103). Also, other factors, like phytochemicals and fiber, also played a significant role in renal protection by consuming whole food from plant-based diets as well as other components of plants. Thus, it is recommended to incorporate high-quality plant proteins for renoprotective effects.

### Functional Properties of Plant-Based Proteins

Plant proteins have also been utilized as functional foods. A large number of studies have been done to examine and reduce the risk factors of cardiovascular disease, modulating inflammation and immune system by functional analysis and bioactive properties of soy protein (104). The recent systematic review has focused on the bioactive properties of sources of plant proteins, such as rice, lentil, fava bean, pea, lupin, hemp, and oat (105). Various trials have been done to test the benefits of proteins derived from plants by observing the concentrations of insulin, blood glucose, and hormones regulating the appetite. However, conflicts in results were seen when the study was conducted for determining the beneficial effects of plant proteins on postprandial glycemia regulation. A number of components present in plants, like flavonoids and carotenoids, also confer the benefits of bioactive functionality on human health.

In addition to the nutritional quality of plant proteins and their bioactive properties, these compounds also have functional properties. They play a major role in food processing and formulation, i.e., the production of gluten-free and protein-rich food (106). Chemical and physical properties of protein help during the storage, consumption, processing, and preparation of food products. Properties like solubility of the protein, foaming capacity, absorbing capacity of water and fat, foam stability, gel-forming, and emulsifying activity are involved in protein interaction by combining with other molecules, like proteins, carbohydrates, salts, lipids, water, and volatiles. These functional properties are largely affected by the molecular size of peptides and/or proteins, charge distribution, and structure of the protein. Additionally, different environmental conditions that affect the structural changes of protein during food processing will also affect the functional properties of plant proteins (107). For improvement of nutritional quality and potential health benefits, different protein formulations can be added, such as isolates, concentrates, and protein flours. However, the functional properties of various plant-based proteins were utilized in the industrial production of food products. Briefly, various functional properties such as protein solubility during beverage production lead to solvation of protein; absorption of water molecules and their binding allows entrapment of water in bread, meat, cakes, sausages, etc.; absorption of fat is linked with binding of free fat in meats, doughnuts, and sausages; emulsifying properties of proteins lead to the production and stabilization of emulsions of fats in pasta, cakes, sausages, soups, etc.; protein's foaming properties permit the entrapping of gasses by forming stable films in whipped toppings, bakery products, cakes, and desserts; gelation properties are linked with the formation and maintaining of protein matrix in meats, cheese, and curds (106).

### Applications of Plant-Based Proteins in Food and Non-Food Industries

Proteins are the important ingredients of the human diet with great complexity and diversity that play an important role in structural and functional development (29, 49). Plant protein provides many essential amino acids, vital macronutrients and is sufficient to achieve full protein nutrition. Moreover, plants have a high demand for the supply of protein to the increasing population (12). Thus, instead of animals, plants were considered the bioproduction system for useful substances, especially in medicine, which usually provide a large number of secondary metabolites having therapeutic effects. These substances produced by plants mainly help to protect from predators and pathogens, attract pollinators, and have properties like anti-inflammatory, wound-healing, anti-microbial, psychoactive, etc. (1), and hence utilized for protecting and maintaining human and animal health.

Different sources of plants have been widely used as supplements of protein, such as cereals (wheat, rice, millet, maize, barley, and sorghum), legumes (pea, soybean, bean, faba bean, lupin, chickpea, and cowpea), pseudocereals (buckwheat, quinoa, and amaranth), nuts and almonds, and seeds (flaxseed, chia, pumpkin, sesame, and sunflower). Along with providing health benefits, proteins also play a significant role in food formulations because of their diverse functions, such as emulsifying, gelling, and thickening agents, and also have water-holding, foaming, and fat absorption ability (16, 17). In addition, these crops have number of beneficial effects on health and have technological and functional properties with industrial applications in development of food. Thus, these proteins play an important role in circular production systems.

Food derived from plants plays a vital role in human health as an important source of bioactive components, minerals, vitamins, and bioactive peptides (4). In addition, protein obtained from plants provides essential amino acids and improves the overall nutritional status of human diets.

From the last few years, much interest has been paid to search for protein sources with high nutritional quality and functionality in food processing and industrial applications (emulsification, solubility, gelation, foaming, and viscosity oil-holding and water-holding capacities). Recently, the importance and benefits of proteins derived from plants have been trending to provide various health benefits. Many studies have been conducted on the potential impact of dietary proteins derived from plants on reducing cardio-metabolic risk factors, metabolic syndrome, weight management, and obesity (86–88). Most of these studies concluded that there should be an addition of proteins of plant origin in the human diet for reducing the risks associated with cardiovascular disease and metabolic diseases (86). Another interesting area of research to examine the benefits of intake of plant proteins instead of animal protein is reducing cancer risk factors.

Food products containing plant proteins have also been known as functional foods. Various trials have been conducted to test the health benefits of plant-based proteins by observing the concentrations of insulin, blood glucose, and hormones regulating the appetite. Most of the studies were also associated with the intake of plant protein sources and mortality. In a recent cohort from the NIH-AARP (National Institutes of Health-American Association of Retired Persons) Diet and Health Study, the authors also observed the effect of choice of dietary protein on mortality (89). The diet, which is lower in vegetables, fruits, healthy oils, and dairy food, but higher in total protein foods, total grains, saturated fats, sodium, and added sugar, has been under trials to know the differences that help to cure chronic disease, especially chronic kidney disease (CKD) (97). Recent studies have suggested that, along with the amount of protein, protein's origin (for example, plant vs. animal) might be a factor that affects the function of the kidney (98).

In addition to the nutritional quality of plant proteins and their bioactive properties, they play a major role in food processing and formulation, i.e., the production of gluten-free (GF) and protein-rich foods (106). In addition, however, the functional properties of various plant-based proteins were utilized in the industrial production of food products. Various applications, like protein solubility (bread, meat, cakes, sausages, doughnuts, and sausages; emulsifying properties emulsions of fats in pasta, cakes, sausages, soups, etc.; protein's foaming properties, bakery products, cakes, and desserts; and gelation properties provide stability to the protein matrix in meats, cheese, and curds (106).

Some traditional proteins from plant origin have been utilized by humans as a protein source, such as beans, pea, and soybean. Still, various recent studies have been done for novel (such as proteins from insects and algae) (2) and unconventional and alternative protein sources (like agroindustry by-products from extraction of edible oil) (7).

Gluten-free pseudocereals help in curing of patients with celiac disease (35). The food industry helps produce high-quality plant-based milk, egg, and meat analogs, such as sausages, burgers, ground meat, and nuggets. The proteins derived from plants are considered important and functional ingredients with different roles in food formulations, including gelling and thickening agents, foams and emulsions stabilizers, and binding material for water and fat. Most of the proteins have biological activities, like ACE inhibitory, antioxidant, antimicrobial, and stimulating characteristics (70), and the protein from vegetables is also utilized for synthesizing and extracting bioactive peptides.

## Health Issues Linked With Plant-Based Proteins

### Antinutrients

There are many health concerns linked with a large intake of dietary proteins derived from plants. Antinutrients, such as tannins, phenolics, saponins, phytates, glucosinolates, and erucic acid, are naturally produced by plants and further interfere with absorption, digestion, and utilization of nutrients present in food, with other side effects as well (108). The adverse effects of antinutrients might be maldigestion of proteins (protease and trypsin inhibitors), carbohydrates (alpha-amylase inhibitors), autoimmune and leaky gut (e.g., some saponins and lectins), malabsorption of minerals (oxalates, phytates, and tannins), inflammation and interfering in thyroid iodine uptake (goitrogens), behavioral effects, and gut dysfunction (when converting cereal gliadins to exorphins) (108). These adverse effects of antinutrients are generally seen in animals when consumed unprocessed proteins of plant origin. However, these antinutrients also showed beneficial health effects. For instance, at a lower level of lectins, phytates, enzyme inhibitors, saponins, and phenolic compounds, there is a reduction in plasma cholesterol, triglycerides, and blood glucose levels (108). Saponins may play a significant role in liver functioning and decrease platelets agglutination. In contrast, some of the saponins and also protease inhibitors, phytates, phytoestrogens, and lignans might help in reducing cancer risk (108). Additionally, tannins also have antimicrobial effects (108). To reduce the concentration of antinutrients in plant proteins and their adverse effects, various treatment processes, such as fermentation, soaking, gamma irradiation, sprouting (germination), heating, and genomic technologies, have been adopted (108). Food processing techniques also remove most of the antinutrients like phytates, glucosinolates, erucic acid, and also insoluble fiber from canola proteins that further improve and increase the digestibility and bioavailability (109).

### Isoflavones and Soy Protein

Soy protein is associated with both positive and negative health concerns. The adverse effect on health is due to the presence of isoflavones in soy protein, which are chemically similar to estrogen and could also be bound to estrogen receptors (110). Due to soy isoflavones, the issue of endocrine-disrupting effect is seen on thyroid and reproductive hormones at higher doses in rodent and *in vitro* cell culture studies (111–113). The isoflavones content of different ingredients of soy protein has been reported; for example, isolates of soy protein (88–164 mg/100 g), defatted and whole soy flours (120–340 mg/100 g), textured soy protein isolates that are commercially used (66–183 mg/100 g), and soy hypocotyl and flours' commercial isolates (542–851 mg/100 g) (114). Therefore, consumers mainly avoid taking soy proteins due to various adverse effects on thyroid and reproductive hormones. The study conducted by the European Food Safety Authority in 2015 showed that 35–150 mg daily doses of isoflavones in pre- and postmenopausal women resulted in no significant enhancement in breast cancer risk, uterus's histopathological changes or thickness in the endometrial lining of the uterus, and thyroid hormonal status for about 30 months (115). A meta-analysis has also been done on 15 men of different ages and found that intake of 60 g/day of soy protein has not been linked with sex hormone-binding globulin, changes in testosterone, free androgen index, or free testosterone (116). Also, it did not influence the parameters of semen quality, such as sperm concentration, semen volume, sperm mobility, sperm count, sperm percent motility, sperm morphology, and total motile sperm count in healthy men (117). It has also been reported in the meta-analysis that intake of soy protein might be linked with reducing breast cancer risk in women (118–120).

### Plant-Based Proteins and Their Association in Allergenicity

There is an increasing trend of consuming plant proteins, which indicates that different sources of protein from plants influence our health. Such dietary proteins may also have some adverse effects, including allergenicity. An allergy from food is basically an adverse effect that results inactivation of immune response when exposed to a food. According to the literature review, food allergy is found to affect up to 10% of the population (121). It has been identified that more than 170 foods in the United States of America are responsible for food allergies. Foods commonly causing allergy are tree nuts, soy, wheat, fish, peanuts, milk, shellfish, and egg. Other common food allergens based on the countries are lupines (European Union); sesame seeds (Canada, European Union, and Australia); buckwheat (Japan and Korea), and mustard (European Union and Canada) (122). A higher number of children than adults are sensitive to dietary proteins that mainly cause allergy (123).

Food allergens from plants are mainly categorized into four families, the cupin superfamily, the prolamin superfamily, profilins, and the Bet v 1 family. More than 50% of allergens of plant proteins fall into two categories, i.e., the cupin and prolamin superfamilies (124). The prolamin family has 8 cysteine residues of amino acid that is conserved with pattern CXnCXnCCXnCXCXnCXnC, which mainly stabilizes the structure of protein and contributes proteins allergenicity. The most commonly found allergens are cereal prolamins, α-amylase, 2S albumins, non-specific lipid transfer proteins, and trypsin inhibitor, protein families.

## Comparison Between Animal and Plant-Based Proteins

Dietary proteins could be derived from animals and plants. Animal protein, although higher in demand, is generally considered less environmentally sustainable. A gradual transition from animal to plant-based protein food may be desirable to maintain environmental stability, ethical reasons, food affordability, greater food safety, fulfilling higher consumer demand, and combating of protein-energy malnutrition. Since the last 20 years, among the alternative sources of protein, the scientific research team and private companies have mainly focused on algae, earthworm or earthworm meal, insects, and other invertebrates (52, 53). Nowadays, food derived from plants plays a vital role in the human diet as an important source of bioactive components, such as vitamins, phenolic compounds, or bioactive peptides. Hence, these components are very helpful to human health and protect against various pathogens (4). Instead of animals, plants were considered the bioproduction system for useful substances, especially in medicine, which usually provide a large number of secondary metabolites having therapeutic effects. These substances produced by plants mainly help protect from predators and pathogens, attract pollinators, and have properties like anti-inflammatory, wound healing, anti-microbial, psychoactive, etc. (1), and hence utilized for protecting and maintaining human and animal health.

The proteins derived from plant-based foods are increasingly used as a health-promoting and economical alternative source in place of animal proteins in human nutrition. However, various limitations, such as increased cost, limited supply, biodiversity loss, hazard for human health in different diseases, freshwater depletion, and susceptibility to climate change, replace animal-based proteins (5–7). Moreover, it is hard and expensive to extract an adequate amount of animal proteins; therefore, an alternative for improving the nutritional status of humans is mainly received from plant proteins.

Globally, protein is produced from both plants (80%), such as cereal grains, beans, soy, pulses, nuts, vegetables, and fruits, as well as animals (~20%) in the form of meats, milk, eggs, fish, yogurt, and cheese (50). Compared to animal-based proteins, the proteins derived from plant-based foods are rich in fiber, polyunsaturated fatty acids, oligosaccharides, and carbohydrates. Therefore, they reduce the cardiovascular diseases and type II diabetes (8). Increased urbanization and economic development have led to various transitions in dietary patterns in the population of low- and middle-income countries, especially the demand for foods derived from animals, which was seen in developing countries.

Recently, plant-based sources of protein have dominated the supply of proteins throughout the world (57%), with the remaining 43% consisting of dairy products (10%), shellfish and fish (6%), meat (18%), and other products from animals (9%) (3, 19). Generally, the daily intake of protein is provided by animal-based foods. However, changes in the consumers' requirement led to adoption of alternative sources of proteins for human consumption. Therefore, emerging factors for animal proteins like growth of world population, climate change, and production of protein sources that are economically and environmentally sustainable need more research focus, and that is mainly dedicated to proteins from plants with high content, resilient to changing of climate and providing balance nutrition in humans' diet.

Compared with animal-based protein, the proteins derived from plants are easier to produce. Still, when utilized as dietary sources for human consumption, most plant proteins are deficient in essential amino acids and are, therefore, nutritionally incomplete. For example, some cereal proteins are low in tryptophan, lysine, and threonine content. In contrast, vegetable proteins and legumes have lower sulfur-containing amino acids, such as methionine and cysteine (58). Due to this deficiency, these essential amino acids become the limiting factor in legumes and cereals. Practically, neither legumes nor cereals can compensate for the deficiency of amino acids for other crops, and, hence, feed diets regularly provide supplementary amino acids.

Many studies have been done on the potential impact of dietary proteins derived from plants and serve as reducing cardio-metabolic risk factors. The first study for the synthesis and intake of plant proteins as an alternative to animal protein was reported (15). However, the regulation of protein intake is critical to many physiological development and functions. Therefore, enhancing the proteins derived from plants in adolescent diets as a substitute for animal-based proteins help in controlling obesity and other cardio-metabolic factors (85). Although plant-based diets are mainly linked with reducing the risk of diabetes (91), it is not clear that substituting the plant-based proteins for animal proteins helps in reducing the risk of diabetes in the population. After studying and analyzing the Nurses' Health Study II dataset, Malik et al. (92) observed that 5% substitution of vegetable protein for animal protein was linked with the 23% reduction of type 2 diabetes risk. Another interesting area of research to examine the benefits of the intake of plant proteins instead of animal protein is reducing cancer risk factors (96).

## The Modification Approaches of Plant-Based Food Proteins

Protein modification is the process of alteration of the chemical groups or molecular structure of a protein by specific methods for improving the bioactivity and functionality of proteins. The modification approaches for plant-based proteins help them to make multifunctional food products. The modification of proteins can be classified into physical (18, 62, 69–82), chemical (125–130), biological (131, 132), and other novel methods (133–137) as briefly described in Tables 1–3. The physical modification approaches include heat treatment (such as conventional thermal treatment, ohmic heating, microwave heating, radio frequency treatment, infrared irradiation), gamma irradiation, electron beam irradiation, ultraviolet radiation, pulsed-electric field, high-pressure treatment (such as high hydrostatic pressure, dynamic high-pressure fluidization), sonication, extrusion, ball mill treatment, cold atmospheric plasma processing, and ultrafiltration. The chemical modification approaches include glycation, phosphorylation, acylation, deamidation, cationization, and pH shifting treatment. The biological modification approaches include enzymatic modification and fermentation. Instead of physical, chemical, and biological modifications, various other modification approaches have been identified, which include complexation (such as protein-polysaccharide, protein-protein, protein-phenolic, and protein-surfactant) and amyloid fibrillization (Tables 1–3) (138).

**Table 2:** The commonly used chemical modification methods of protein and their applications.

**Modification methods**		**Description/Applications**	**References**
Chemical modification	Glycation	Improve protein functionalities, emulsifying ability, solubility of the protein, foaming ability, thermal stability, and flavor profile	(125)
		Reduce beany flavor in some plant-based proteins	
		Having strong immunomodulatory properties	
	Phosphorylation	Keep nutritive bioavailability	(126)
		Improve solubility, thermal stability, viscosity, viscoelasticity, thermal aggregation functional, foaming, and emulsifying properties	
		Increase *in-vitro* digestibility	
	Acylation	Improve solubility, emulsifying, foaming and functional properties, emulsion stability, and water holding capacity	(127)
		Increasing the molecular weight of some proteins and hydrophobicity will led to improvement or enhancement of thermal stability and gelling properties	
	Deamidation	Mask the bitterness	(128)
		Improve techno-functionality, solubility, water holding capacity, emulsifying, and foaming properties	
		Reduce beany flavor, grittiness, and lumpiness	
		Decrease the allergenicity of plant-based proteins	
	Cationization	Modify techno-functionality	(129)
		Improve solubility, encapsulating, and emulsifying properties	
	pH shifting treatment	Change the structural and functional properties of proteins	(130)
		Improve extensibility and tensile properties of the formed films and also the functionality, such as enhanced solubility, surface hydrophobicity, antioxidant activity, rheological, foaming, and emulsifying ability	
		Induce protein reactivity by promoting its unfolding	

**Table 3:** The commonly used biological and some other modification methods of protein and their applications.

**Modification methods**			**Description/Applications**	**References**
Biological modification	Enzymatic modification		Improve emulsifying ability, techno-functionality, protein solubility, antioxidant ability, interfacial properties, foaming ability, oil holding capacity, and bioactivity of plant-based proteins	(131)
			Increase the hydrophobicity and surface-active properties of the generated hydrolysates	
			Decrease the bitterness	
	Fermentation		Improve protein solubility, water and oil holding capacity, foaming, and functional properties	(132)
			Promote nutritional and antioxidant properties and also the digestibility	
			Degrade allergens and anti-nutritional compounds	
			Decrease immunoreactivity, bitter, and beany off-flavors of different plant-based proteins	
Others	Complexation	Protein-polysaccharide	Modulate techno-functional properties and address issues such as physical stability around their isoelectric point	(133)
			Improve solubility, susceptibility, stability, emulsifying, and foaming properties	
			Reduce the bitterness of potato protein	
		Protein-protein	Improve techno-functionality	(134)
			Increase water solubility	
		Protein-phenolic	Exhibit different biological activities such as antioxidant, antimicrobial, anticancer, antiallergenic, anti-inflammatory, and also higher thermal stability	135
			Polyphenolic compounds reduce solubility of plant-based proteins	
		Protein-surfactant	Tune the amphipathic properties by modulating hydrophobic or hydrophilic degrees	(136)
			Improve encapsulation efficiency, physicochemical properties, solubility, emulsifying and foaming properties, water dispersibility, pH, salt, physical-, photo-, acid-, and thermal stability	
			Increase stability	
	Amyloid fibrillization		Improve protein functionalities in different applications such as drug and nutraceutical delivery platforms	(137)
			Increase surface hydrophobicity	
			Improve foam, emulsion Pickering stabilizers, degradable films, ultralight aerogels, gels, water purification filters, and rheological properties	

## Protein Extraction Technologies

**Figure 4:**
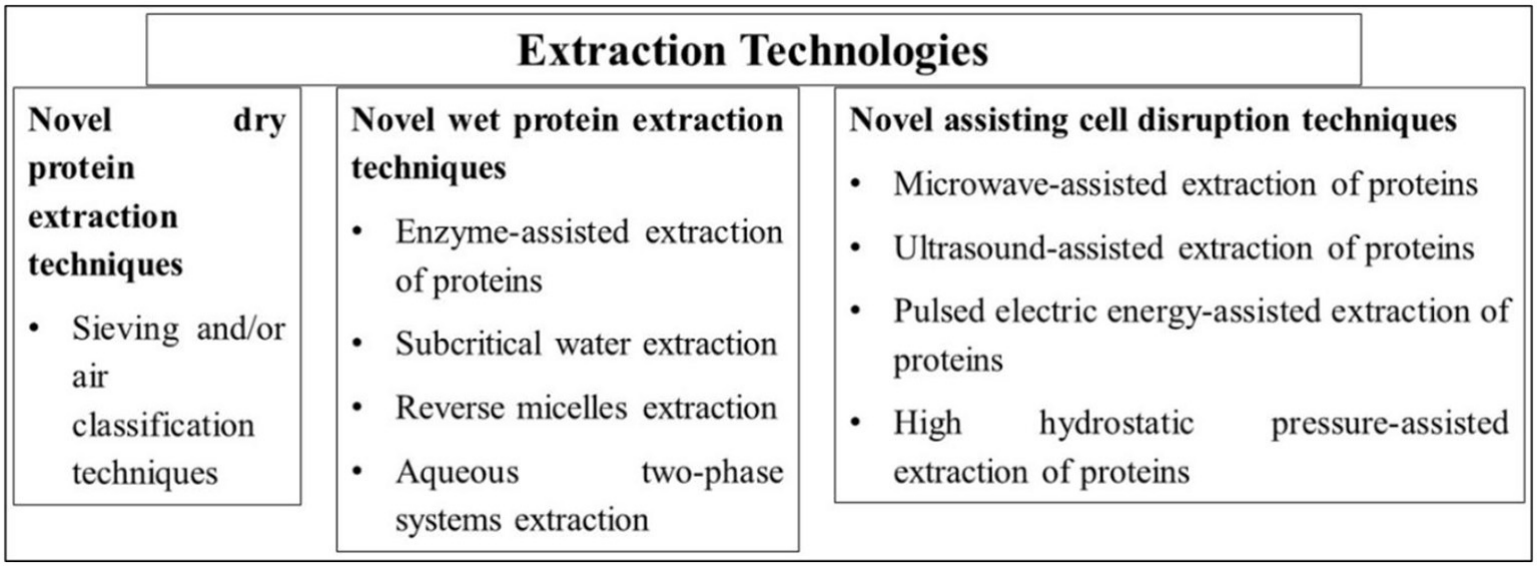
Extraction technologies for plant-based proteins.

The advancement in recombinant technologies of protein production, such as engineering of expression hosts, upstream cultivation optimization (e.g., nutritional, bioreactor design, and physical parameters), and development of protein extraction methods supported the growth of the market (7, 13). The use of protein extraction technologies can help improve the yield of extracted protein and its nutritional and functional properties. Hence, a suitable type of protein extraction method should be selected (Figure 4).

### Dry Protein Extraction Technique

Sieving and/or air classification techniques, majorly a part of novel dry protein extraction techniques, have been widely used to prepare fiber or protein-rich fractions. Although a high protein yield was generated, however, it utilized more energy than wet protein extraction. Also, the disadvantage of these processes includes the presence of impurity and particle agglomeration (7).

### Wet Protein Extraction Technique

In wet protein extraction techniques, the process starts with protein solubilizing in a medium with the pH far from the isoelectric point and then precipitating in that medium where pH is close to the isoelectric point. Several protocols for acidic and alkaline extraction of protein have been reported (7).

#### Enzyme-Assisted Extraction of Proteins

This method is based on the principle of cell wall disruption with specific enzymes that degrade celluloses, hemicelluloses, and/or pectin, and also proteases that help in the hydrolyzation of protein for solubility enhancement. With the degradation of cell walls, protein bodies released are enabled. This method needs more processing time, high cost, more energy consumption, and suitable conditions like temperature and pH. Still, this method is mostly used with lower environmental impact and superior quality of products for human consumption (7, 139).

#### Subcritical Water Extraction

In this technique, hot water in the range of 100–374°C with high pressure (for maintaining it into the liquid state) has been used. Biomaterials like carbohydrates and proteins have been hydrolyzed by this method without using an additional number of catalysts. For example, when soy meals were heat denatured, soy protein extraction yield was significantly increased with this method by 59.3% (139).

#### Reverse Micelles Extraction

This method applies reverse micelles—surfactant molecules aggregate of the nano-meter size that generally contains inner cores of water molecules inside non-polar solvents. The polar molecules of water present in reverse micelles help in solubilizing hydrophilic biomolecules like proteins. The three-phase system called a water–surfactant–organic solvent system has been formed by reverse micelles to protect the denaturation of proteins by organic solvents inside the polar water pools, using forward extraction or backward extraction (7).

#### Aqueous Two-Phase Systems Extraction

This extraction method is formed when two polymers like two salts or one salt and one polymer are mixed in a suitable concentration at a particular temperature. This method has been considered as the environment-friendly method of protein extraction. It was first reported by Zeng et al. (140) for extracting proteins by an ionic liquid aqueous two-phase system, resulting in proteins extraction with a yield of 99.6% (7).

### Novel-Assisting Cell Disruption Techniques

Cell disruption is the initial process in both dry and wet techniques of protein extraction, which helps release protein from protein bodies. Previously, cell disruption was done by mechanical methods like milling, grinding, etc., or chemical or thermal treatments.

#### Microwave-Assisted Extraction of Proteins

This technology utilizes electromagnetic radiations having a frequency between 300 MHz and 300 GHz, which helps in hydrogen bond disruption, dissolved ions migration, and enhancement of porosity of the biological matrix, which leads to the extraction of protein. For example, one study reported the utilization of this technique to extract proteins from rice bran (141).

#### Ultrasound-Assisted Extraction of Proteins

This technology utilized sound waves, having a frequency of 20 kHz that induces the phenomenon of cavitation, which enhances the matrix porosity and improves solvent permeation into the matrix. This method has the advantage of effective mixing, selective extraction, faster energy transfer, reduced extraction temperature and thermal gradients, faster response, reduced equipment size, and increased production. Yet, denaturation of protein structure and disruption of functional properties of proteins are reported (7).

#### Pulsed Electric Energy-Assisted Extraction of Proteins

Several pulsed electric energy technologies for proteins extraction have emerged. This method uses electric pulses of short duration (from several nanoseconds to several milliseconds) of high-pulse amplitude (from 100 to 300 V/cm to 10–50 kV/cm) for the induction of structural changes of the compound of interest. Among a large number of PEE techniques, pulsed ohmic heating (POH), pulsed electric fields (PEF), and high-voltage electrical discharges (HVED) have been widely used in the food industry (7).

#### High Hydrostatic Pressure-Assisted Extraction of Proteins

High hydrostatic pressure-assisted extraction of proteins is mostly used in the food industry for large-scale microbial cell disruption, meat tenderization, and emulsification. This method is only restricted to bioactive compounds instead of proteins. However, with the application of several HHP iterations, the efficiency of separation and extraction yield has been reduced due to swelling of the cell wall, increase in dynamic viscosity, and size of the particle (7).

## Issues, Challenges, and Future Prospects of Plant-Based Proteins and Their Utilization in Food Products

The proteins derived from plants are considered as important and functional ingredients, having different roles in food formulations as gelling and thickening agents, foams and emulsion stabilizers, and binding material for water and fat (142–145). Most of the proteins have biological activities, like antihypertensive, antioxidant, antimicrobial, and stimulating characteristics (146, 147), and the protein from vegetables is also utilized for synthesizing and extraction of bioactive peptides (148, 149). However, most of the proteins from plant origin are interactable because of their susceptibility and complexity of ionic strength, pH, and temperature, and also have poor water solubility that mainly limits the applications of plant-based proteins (150). Most of the plant-based proteins, like flaxseed, soy, and pea proteins, have the combined nature of various proteins with different fractions, and, hence, they have a wide range of isoionic point (pI). Therefore, modulating the properties of plant-based proteins for improving their functions and formulation characteristics is essential. A deep understanding of the functional and physicochemical properties of proteins derived from plants is necessary for improving their utilization in food formulation and nutritional value (151–153). The presence of some particular plant residues considered as antinutrients is another challenge of plant-based proteins. These compounds are produced in plants having various biological properties, such as they protect the plants and seeds from insects, fungus, viruses, and other microbes. Therefore, some of the modification approaches discussed have been used to reduce or eliminate the adverse effects of antinutrients (18). Furthermore, some plant-based proteins have challenges in food applications due to their bitter taste, which can be masked by various modulation techniques. The methods of modification for plant-based proteins should be carefully chosen, especially in pharmaceutical and food applications, because these methods have effects on the organoleptic and functional characteristics and nutritional value of plant proteins.

The bio-efficacy of any active compounds generally depends on various factors, like digestibility, solubility, bioaccessibility, food matrix, transporters, metabolizing enzymes, and molecular structures. Therefore, identifying the bioavailability of food constituents is challenging. There are many challenges associated with sustainability and food availability that needs to be solved with different methods of protein modification. The higher amount of essential nutrients found in animal products (meat, milk, egg, etc.) was important and provided a large number of nutrients in the daily diet compared with plant-based proteins (154). Although animal-meat-based products provide a large nutrient component, however, the disease associated with animals, unhygienic conditions, and environmental impact will all provide more attention to the plant-based proteins. Because of that, consumers are also more focused on the health and environmental benefits of plant-based diets, promote the food guidelines on the basis of health and sustainability criteria, produce more attractive plant-based alternative products, and realign their fiscal policy along with environmental and efficiency criteria (155–159).

## Conclusions

People are facing protein and mineral deficiency in their diet throughout the world, especially in developing countries. This challenge is due to lower consumption of pulses and cereals in their diets and other foods that are rich in zinc, iron, calcium, and magnesium. These foods derived from plants also contain higher levels of antinutritional factors that bind to the minerals ions and reduce bioavailability and absorption of plant minerals as well as proteins. Animal protein, although higher in demand, is generally considered less environmentally sustainable and prone to disease conditions, which negatively impact health. A gradual transition from animal- to plant-based protein may be desirable in order to maintain environmental stability, ethical reasons, affordability of food, greater food safety, fulfilling higher consumer demand, and combating of protein-energy malnutrition. Nowadays, products made with proteins from plant origin gain popularity throughout the world. Plant-based proteins have been linked with a number of health-related functionalities. Plant-based proteins are becoming innovative and fast-growing ingredients in various food application industries due to a large number of benefits over animal-derived proteins. Various technologies help in improving the functional and nutritional properties of plant-based proteins. Generally, plant-based proteins have inferior functionality as compared with animal proteins, and also various factors affect their nutrient quality; hence, modification approaches have been required. Different physical, chemical, biological, and other approaches were also mentioned for modification of proteins that induce the structural, chemical, and biophysical changes in protein from plant origins.

This review mainly focuses on the current state of using plants for the production of protein. The potential plants offering various sources and their alternative with high-quality protein demand for future consumption were discussed. Factors that affect protein consumption, bioavailability, and also protein production techniques were covered. Various bioactive and functional properties of plant-based proteins, as well as the factors affecting the nutritional quality of plant-based proteins and the future research strategies, were explained. The modification approaches, protein extraction, purification technologies, along with digestibility, absorption, and bioavailability of plant-based proteins, were discussed. Finally, it gave an idea of issues and challenges as well as future prospects in this emerging area.

## Author Contributions

SL conceived the idea. SL and FK wrote the manuscript. PY, ZD, RS, and AK edited the manuscript. All authors contributed to the article and approved the submitted version.

## Conflict of Interest

The authors declare that the research was conducted in the absence of any commercial or financial relationships that could be construed as a potential conflict of interest.

## Publisher's Note

All claims expressed in this article are solely those of the authors and do not necessarily represent those of their affiliated organizations, or those of the publisher, the editors and the reviewers. Any product that may be evaluated in this article, or claim that may be made by its manufacturer, is not guaranteed or endorsed by the publisher.
